# Liposomes: structure, composition, types, and clinical applications^☆^

**DOI:** 10.1016/j.heliyon.2022.e09394

**Published:** 2022-05-13

**Authors:** Hamdi Nsairat, Dima Khater, Usama Sayed, Fadwa Odeh, Abeer Al Bawab, Walhan Alshaer

**Affiliations:** a Pharmacological and Diagnostic Research Center, Faculty of Pharmacy, Al-Ahliyya Amman University, Amman, 19328, Jordan; b Department of Chemistry, Faculty of Arts and Science, Applied Science Private University, Amman, Jordan; c Department of Biology, The University of Jordan, Amman, 11942, Jordan; d Department of Chemistry, The University of Jordan, Amman, 11942, Jordan; e Hamdi Mango Center for Scientific Research, The University of Jordan, Amman, 11942, Jordan; f Cell Therapy Center, The University of Jordan, Amman, 11942, Jordan

**Keywords:** Liposomes, Phospholipids, Lamellarity, Stealth liposomes, Vaccinations

## Abstract

Liposomes are now considered the most commonly used nanocarriers for various potentially active hydrophobic and hydrophilic molecules due to their high biocompatibility, biodegradability, and low immunogenicity. Liposomes also proved to enhance drug solubility and controlled distribution, as well as their capacity for surface modifications for targeted, prolonged, and sustained release. Based on the composition, liposomes can be considered to have evolved from conventional, long-circulating, targeted, and immune-liposomes to stimuli-responsive and actively targeted liposomes. Many liposomal-based drug delivery systems are currently clinically approved to treat several diseases, such as cancer, fungal and viral infections; more liposomes have reached advanced phases in clinical trials. This review describes liposomes structure, composition, preparation methods, and clinical applications.

## 1 Introduction

Drug delivery systems (DDSs) offer the potential to enhance the therapeutic index of drugs by increasing the drug concentration, the residence time in target cells and minimizing the side effects [1]. DDSs involve delivering the potentially active drug to the site of action *via* a nano-vehicle to enhance the pharmacological properties of free drugs and cover their undesirable features through improving drug pharmacokinetics and biodistribution, as well as acting as drug reservoirs [2, 3]. These nanoparticles (NPs) usually ranged from a few nanometers to several hundred nanometers according to their intended application [4]. Different natural, organic and inorganic materials are used to create NPs including ceramic, polymers, metals [4], and lipids that generate nanoparticles like micelles and liposomes [5, 6, 7].

Therapeutic drugs are incorporated into the NPs mainly by physical interactions including, entrapment, surface attachment, or encapsulation [8]. These variations and unique properties of different NPs could be used to improve the characteristics of traditional therapeutics [8]. Nanomedicine facilitates designing novel therapeutic options in the nanoscale range to deliver a variety of active biomedical ingredients for the treatment, prevention, and diagnosis of many diseases [1, 9].

**Figure 1:**
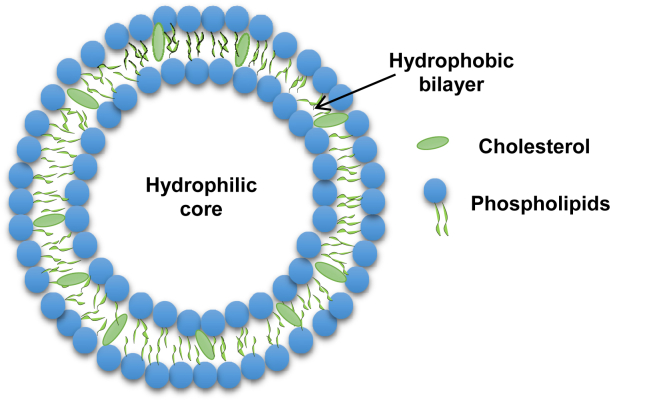
Schematic representation of liposomes.

Despite the fast progress in this field, most nanoparticles-based drug delivery systems show improper loading capacity with a lack of specificity against their targets [10]. As a result, the promising advances in the drug delivery systems should involve designing high and regulated capacity nanocarriers functionalized by recognition ligands that target specifically unique or overexpressed biomarkers [11]. Liposomes are the most explored nanocarriers used in targeted drug delivery systems. Liposomes are spherical lipid vesicles (usually 50–500 nm in diameter particle size) composed of one or more lipid bilayer**s**, as a result of emulsifying natural or synthetic lipids in an aqueous medium [12, 13] (Figure 1). Liposomes were firstly discovered in the 1960's by Bengham and later became among the most expansive drug delivery systems [14]. Liposomes nanoemulsions are widely used nanoparticles in nanomedicine mainly due to their biocompatibility, stability, ease to synthesize and high drug loading efficiency [15, 16], high bioavailability [17], and their safe excipients used in these formulations [18]. Due to their size, hydrophobic and hydrophilic characteristics and their ability to encapsulate drug molecules either in the aqueous interior of the vesicles or in the lipophilic membrane [19], liposomes are considered promising to be used effectively as drug delivery systems. Several liposomal-based drug delivery systems have been approved by Food and Drug Administration (FDA) for disease treatment in the market [20, 21]. Moreover, liposomes are suitable for diagnostic and therapeutic applications using several routes of administration, including ocular [22], oral [23], pulmonary [24], transdermal [25], and parenteral [26, 27, 28]. Liposomes are primarily created from phospholipids such as soybean phosphatidylcholine [29] or synthetic dialkyl or trialkyl lipids [30]. Incorporation of cholesterol into liposomes is indispensable since cholesterol modulates membrane permeability, changes fluidity, and improves the stability of bilayer membranes in the presence of biological fluids such as blood and plasma [31, 32]. Liposomal formulations may also contain polymers [33], and even membrane protein [34] to prolong their circulation half-life, improve the biodistribution profile and enhance the encapsulated drug effectiveness [35] Moreover, Stealth stabilized liposomes, incorporating phospholipids-attached polyethyleneglycol (PEG) into liposomes infrastructure, has been shown to be a useful method for modifying liposomes pharmacokinetic properties and biodistribution profiles [36]. The current review describes liposomes compositions, types, methods of preparation, and clinical applications.

## 2 Liposomes

According to the liposomes structures, they are classified into four categories based on size and number of bilayers: small unilamellar vesicles (SUV), large unilamellar vesicles (LUV), multilamellar vesicle (MLV), and multivesicular vesicles (MVV). Liposomes have mono phospholipid bilayer in a unilamellar structure, while they have an onion-like structure in a multilamellar structure. MVV form a multilamellar arrangement with concentric phospholipid spheres as many unilamellar vesicles are produced within larger liposomes [37]. Liposome encapsulation efficiency increases with liposome size and decreases with the number of bilayers for hydrophilic compounds only [38]. The size of the vesicles is an important factor that controls the circulation half-life of liposomes. Both the size and number of bilayers influence the amount of the encapsulated drug. When liposomes are employed for drug delivery, the desired vesicles usually extend from 50 nm to 150 nm. Liposomes interaction with the cell membrane is represented by various theories: specific (modified with receptor-mediated) or nonspecific endocytosis [39], local fusion (adhesion) [40], phagocytosis [41], and absorption into the cell membrane [42]. Liposome-cell interactions are influenced by a variety of factors, including composition [43], the diameters of liposomes, surface charge [44], targeting ligand on the liposome surface, and biological environment [45].

### 2.1 Liposomes compositions

#### 2.1.1 Lipids and phospholipids used for liposomes

Structurally, liposomes are spherical or multilayered spherical vesicles made by the self-assembly of diacyl-chain phospholipids (lipid bilayer) in aqueous solutions [46]. The bilayer phospholipid membrane has a hydrophobic tail and a hydrophilic head [21, 47] that leads to the formation of an amphiphilic structure. Liposomes can be made from both natural and synthetic phospholipids [48]. Lipid composition strongly affects liposome characteristics that include: particle size, rigidity, fluidity, stability, and electrical charge [5, 49]. For example, liposomes formulated from natural unsaturated phosphatidylcholine, as egg or soybean phosphatidylcholine, provide highly permeable and low stable properties. Though, saturated-phospholipids-based liposomes such as dipalmitoyl phosphatidylcholine led to rigid and almost impermeable bilayer structures [21].

The hydrophilic group in the lipids may be negatively, positively charged, or zwitterionic (both negative and positive charge in the same molecule). The charge of the hydrophilic group provides stability through electrostatic repels. The hydrophobic group of lipids varies in the acyl chain length, symmetry, and saturation [50]. The lipids that used in liposomes preparation may be classified as:

#### 2.1.2 Natural lipids

**Figure 2:**
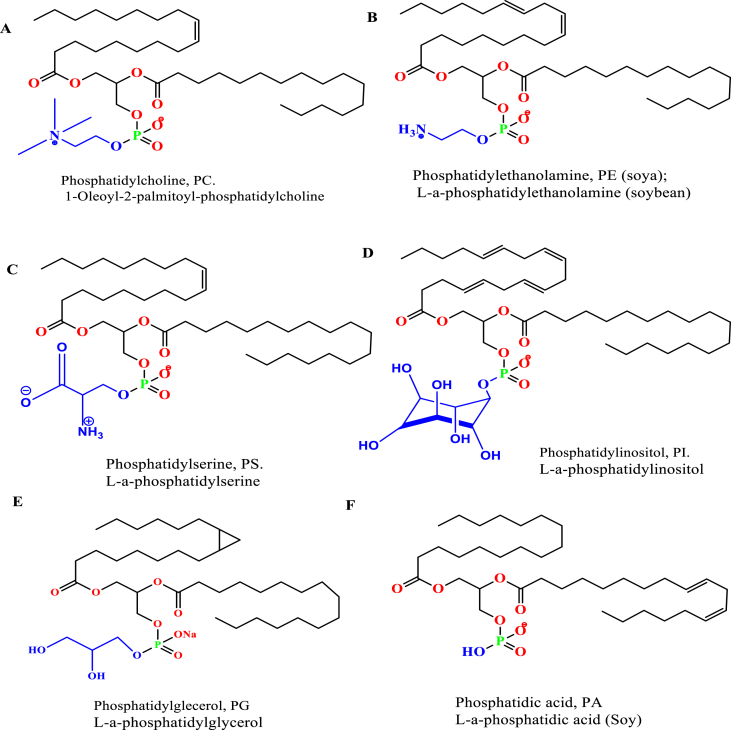
Natural phosphatides the most used to produce liposomes; A) Phosphatidylcholine, B) Phosphatidylethanolamine, C) Phosphatidylserine, D) Phosphatidylinositol, E) Phosphatidylglecerol, and F) Phosphatidic acid.

The membrane bilayer of normal cells are mainly composed of glycerophospholipids. Phospholipids are consist of a glycerol unit that is bonded to a phosphate group (PO_4_^2−^) and to two fatty acid molecules. The phosphate group can be also bonded to small, essential choline organic molecule [21, 51] (Figure 2A). Natural phospholipids can be obtained from various sources such as soya bean, egg yolk [52]. Phospholipids are classified as phosphatidylcholine (PC), phosphatidylethanolamine (PE), phosphatidylserine (PS), phosphatidylinositol (PI), phosphatidylglycerol (PG), and phosphatidic acid (PA) regarding to the polar head groups. Natural phospholipids are less stable than synthetic phospholipids in liposomes preparation due to the unsaturated characteristics of the hydrocarbon chain [53, 54] (Figure 2). Natural phospholipids composed of a variety of fatty acids, one is a saturated fatty acid as palmitic acid (hexadecanoic acid, H_3_C-(CH_2_)_14_-COOH); margaric acid (heptadecanoic acid, H_3_C-(CH_2_)_15_-COOH) and the other is an unsaturated fatty acid (here oleic acid, or 9Z-octadecenoic acid that identified in egg yolk lecithin [55]. The egg derived phospholipids and PCs are made of these fatty acids patterns: palmitic acid (C16:0), stearic acid (C18:0), oleic acid (C18:1), linoleic acid (C18:2), and arachidonic acid (C20:4). These fatty acids are account for about 92% of the total fatty acid composition with a typical presence of the polyunsaturated fatty acids C 20:4 (n-6) and C22:6 (n-3) in egg phospholipids. Egg PC contains about 40% 1-palmitoyl-2-oleoylphosphatidylcholine. The principal saturated acid was stearic in PE and PS, and palmitic in the other lipids. Furthermore, the fatty acid pattern of the soybean derived account for about 95% palmitic acid (C16:0), stearic acid (C18:0), oleic acid (C18:1), linoleic acid (C18:2), and linolenic acid (C18:3) [56]. Since the unsaturated fatty acids of PE, PS, and PC amounted to over 50% of the total acids, they must occur at both the α- and β-positions of the glycerol moiety of these phospholipids [57].

#### 2.1.3 Synthetic lipids

**Figure 3:**
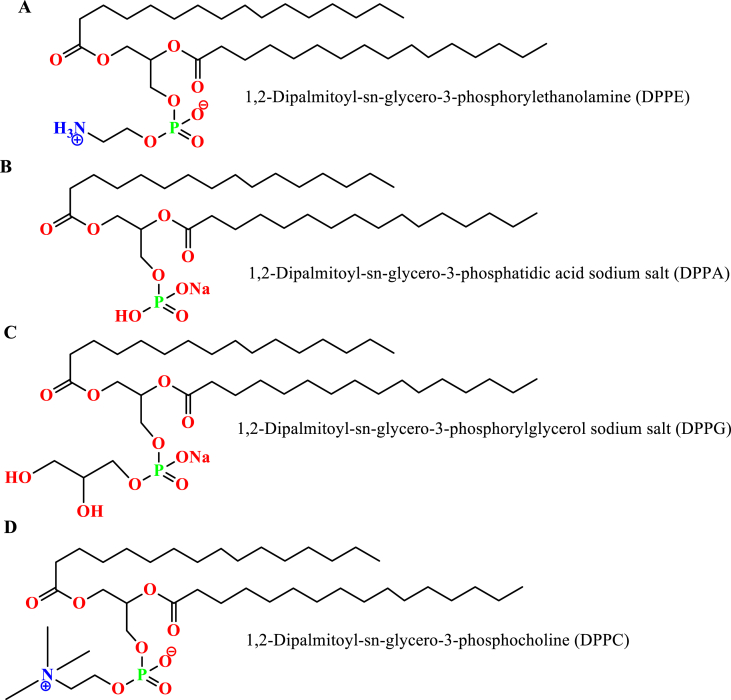
Palmitic acid -based different synthetic phospholipids; A) 1,2-Dipalmitoyl-sn-glycero-3-phosphorylethanolamine, B) 1,2-Dipalmitoyl-sn-glycero-3-phosphatidic acid sodium salt, C) 1,2-Dipalmitoyl-sn-glycero-3-phosphorylglycerol sodium salt, and D) 1,2-Dipalmitoyl-sn-glycero-3-phosphocholine (DPPC).

**Figure 4:**
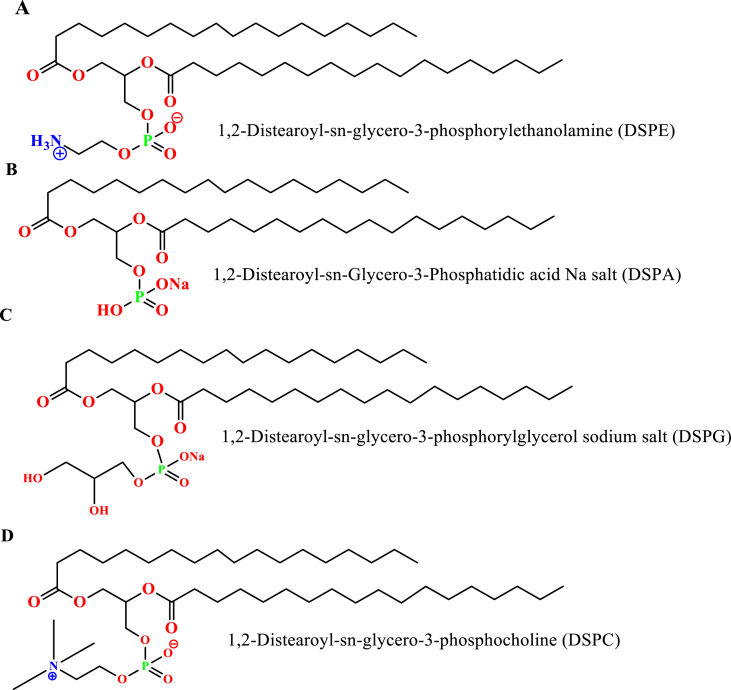
Stearic acid -based different synthetic phospholipids; A) 1,2-Distearoyl-sn-glycero-3-phosphorylethanolamine, B) 1,2-Distearoyl-sn-Glycero-3-Phosphatidic acid Na salt, C) 1,2-Distearoyl-sn-glycero-3-phosphorylglycerol sodium salt, and D) 1,2-Distearoyl-sn-glycero-3-phosphocholine.

Synthetic phospholipids are made by specific chemical modifications to the non-polar and polar regions of the natural phospholipids. The modification enables an unlimited variety of well-defined and categorized phospholipids [56]. The major saturated synthetic phospholipids are based on either using stearic and/or palmitic fatty acid. Figures 3 and 4 represent different possible and commercial, synthetic, and saturated phospholipids usually used to prepare liposomes [53].

**Figure 5:**
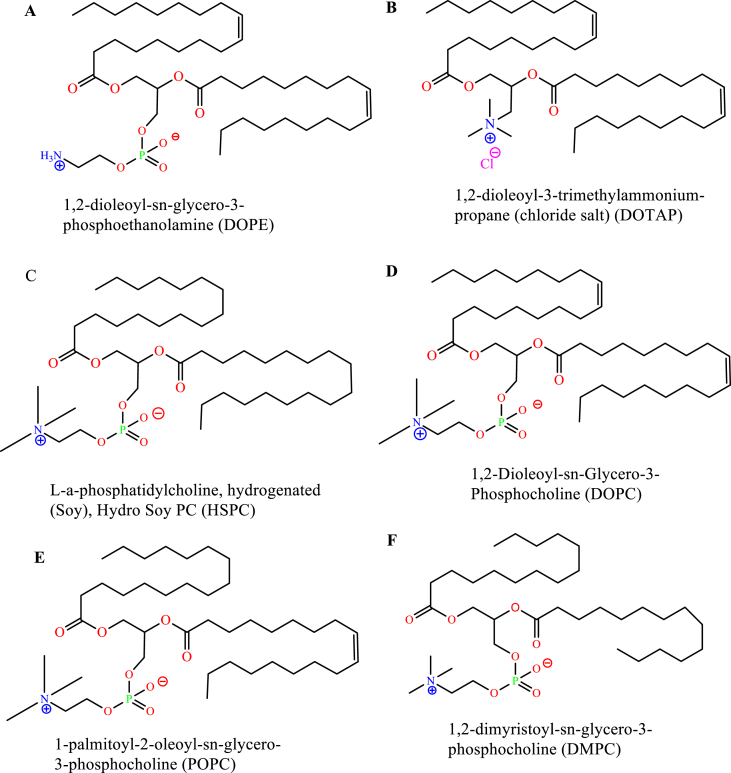
Mixed and different types of synthetic phospholipids; A) 1,2-dioleoyl-sn-glycero-3-phosphoethanolamine, B) 1,2-dioleoyl-3-trimethylammonium-propane (chloride salt), C) L-a-phosphatidylcholine, D) 1,2-Dioleoyl-sn-Glycero-3-Phosphocholine, E) 1-palmitoyl-2-oleoyl-sn-glycero-3-phosphocholine, and F) 1,2-dimyristoyl-sn-glycero-3-phosphocholine.

Additionally, Synthetic phospholipids can be made from mixed fatty acids, unsaturated fatty acids in both hydrocarbons or only in one hydrocarbon chain [52] (Figure 5).

#### 2.1.4 Steroid

**Figure 6:**
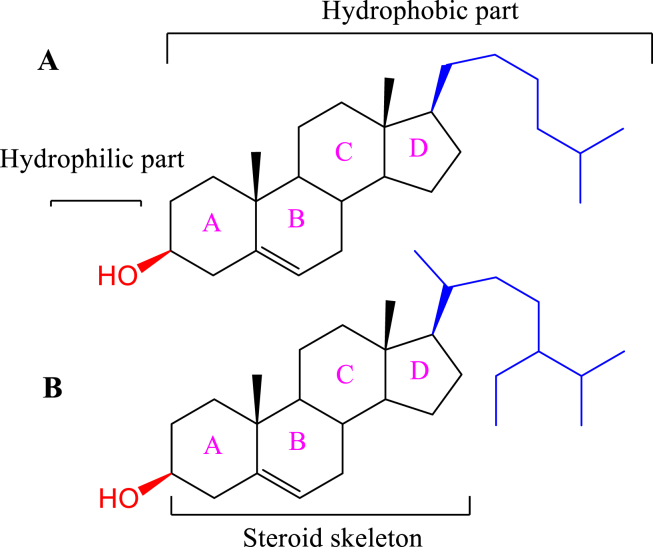
Chemical structure of A) cholesterol, B) β-sitosterol.

Steroid are hydrophobic lipids consists of four-ring structure as shown in Figure 6. Steroid's diversity comes from the various functional groups attached to those rings. Cholesterol is the major steroid usually used in liposomes preparation in a ratio less than 30 % of the total lipids to improve liposomes rigidity and stability since its incorporated in the liposomes lipid bilayer [47, 58]. In a comparative study for cholesterol and β-Sitosterol effect on the liposome membrane features, they found that both steroids reduce liposomes membrane fluidity, increase absolute zeta potential, cause significant changes in particle size, and decrease DPPC phase transition temperature (*T*_m_) and enthalpy [59].

#### 2.1.5 Surfactants

**Figure 7:**
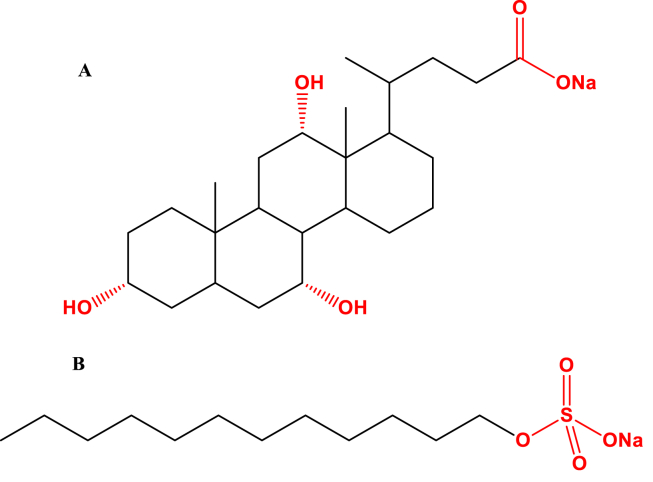
Chemical structure of surfactants A) sodium cholate, B) Sodium dodecyl sulfate (SDS).

Surfactants were utilized in liposomes formulations to modify the encapsulation and release properties of liposomes through surface tension reduction between different immiscible phases [60]. Surfactants are single acyl-chain amphiphiles that destabilize the lipid bilayer of liposomal nanoparticles (Figure 7), thus increasing nano-vessel deformability [61, 62]. Commonly utilized surfactants in liposomes formulations are: sodium cholate, Span 60, Span 80, Tween 60, and Tween 80 [62, 63]. Various surfactants-containing liposomes have been widely used as a carrier in drug delivery to enhance skin penetration of encapsulated therapeutic agents [64]. Ultra deformable liposomes, also called transfersomes, are a Surfactants-based nanovesicles with positive findings in transdermal drug delivery [65, 66]. The key factor making liposomes deformable is edge activator (surfactant). The edge activator can alter the lipid bilayers of vesicles increasing the deformability of them [67]. These nanovesicles are different from conventional liposomes in which they can respond to osmotic pressure by rapid shape transformations only by low energy [67]. Moreover, ultra deformable liposomes showed an increased in the drug transepidermal flow made them more suitable nanovehicle for the topical administration of antihypertensives [68].

### 2.2 Liposomes types

Based on their compositions and applications, liposmes can be classified into conventional liposomes [69], charged liposomes [70], stealth stable liposomes [71], actively targeted liposomes [72], stimuli-responsive liposomes [73], and bubble liposomes [74].

#### 2.2.1 Conventional liposomes

These liposomes were synthesized from natural or synthetic phospholipids with or without cholesterol as a liposomes first generation [75]. Cholesterol was added to improve liposomes fluidity, altering the bilayer rigidity and liposomes stability [31, 32]. Wu et al hat revealed that liposomal membrane rigidity decreased with the addition of cholesterol into a liposomes composed of hydrogenated soybean phospholipids (HSPC) and DSPE-PEG2000. Moreover, liposomes with some rigidity showed excellent tumor penetration and enhanced anti-tumor activity [76]. Kaddah et al investigated the cholesterol content on the permeability and fluidity of DPPC liposome membrane. High cholesterol concentration increased the average liposomes size accompanying with a shape transition from irregular to nanosized, regular and spherical vesicles. In addition, cholesterol induced a decrease in the bilayer fluidity and modulating the release of hydrophilic molecules from lipid vesicles [77]. Jovanović et al reported that increasing cholesterol content in the liposomes decreased the fluidity and enhanced the rigidity of liposomal membranes. They verified that a stable liposome should have an optimal 50 mol %. concentration of cholesterol to obtain and an appropriate membrane fluidity [78]. As a result, cholesterol plays a crucial role in liposomes bilayer fluidity and rigidity, but these properties affected by the cholesterol molar ratio with types of phospholipids used along with the nature of the encapsulated drug. Conventional liposomes showed a short blood circulation time due to their susceptability to elimination by the mononuclear phagocyte system [79] with rapid accumulation in liver and spleen. Hence, MPS obstructs the delivery of conventional liposomes to the target region and restricts their distribution to other tissues of the body [80]. Conventional liposomes also showed relatively limited stability in vitro [81]. As a result, stealth stable liposomes were invented to increase blood circulation and enhanced in vivo liposomes stability [82].

#### 2.2.2 Charged liposomes

Oleic acid and N-[1(2,3-dioleoyloxy) propyl]-N,N,N-trimethylam-monium chloride (DOTAP) are usually used to prepare anionic and cationic liposomes, respectively. Charged liposomes showed higher liposomal stability during the storage, as charged particles repel each other and reduce aggregation abilities. Cationic liposomes are used in gene therapy due to their ability to successfully encapsulate nucleic acids by electrostatic attractions [83].

Cationic liposomes are suitable for delivering various negatively charged macromolecules such as DNA, RNA, and oligonucleotides because their negative charge and rather a large size restrict their passive diffusion into cells [84]. Cationic liposomes also selectively target angiogenic endothelial cells in tumors [85]. Cationic liposomes are considered a potential tool for delivering therapeutics to the brain [6, 86]. Cationic liposomes can cross the BBB by receptor-mediated transcytosis [87] or absorptive-mediated transcytosis [88]. The higher positive charge on the surface of cationic liposomes may affect their blood circulation and lead to due to electrostatic interactions with anionic species in the blood and increase liposomes aggregation that reduces their localization site of action [89, 90]. Decorating the surface of these liposomes with poly ethylene glycol (PEG) protects the them from the circulating proteins, improving the drug efficiency through improving systemic circulation time and decrease immunogenicity [91, 92].

Anionic liposomes are less stable in the bloodstream than neutral and cationic liposomes; they showed a higher clearance rate [44, 93]. Anionic liposomes are usually utilized for transdermal drug delivery because they improve penetration properties through the stratum corneum of the skin [94].

#### 2.2.3 Stealth stabilized liposomes

These second-generation liposomes are characterized by surface decoration with synthetic polymers, glycoproteins, polysaccharides, or specific receptors ligands to achieve narrowed distribution, and accumulation at the intended site [95]. Huayluorinic acid [96], polyvinyl alcohol (PVA), and polyethylene glycol (PEG)) were considered the best model for liposome steric protection. PEGylated liposomes are denoted as stealth liposomes [97, 98]. Doxil® was the first successful pegylated liposome-based product [99]. Stealth stabilized liposomes showed longer circulation time, leading to a better target accumulation than conventional liposomal drugs [100].

#### 2.2.4 Actively-targeted liposomes

Actively-targeted liposomes represent third-generation liposomes. Liposomes' active targeting increases the selectivity of liposome interaction with diseased cells and triggers receptor-mediated endocytosis of the liposome and its payload into the desired cellular target [101, 102].

Many liposomes nanocarriers have been approved for anti-tumor agents delivery by passive ways based on the enhanced permeability and retention (EPR) effect of cancerous cells [103]. Passive targeting does not discriminate between normal and diseased cells [104]; therefore, cell-specific targeting liposomes have been developed to increase the accumulation and localization of anti-tumor agents in diseased cells [104]. Liposomes targeting can be enhanced by incorporating molecular recognition moieties, which can lead to drug transport with better efficacy and low side effects [105]. For example, liposomal targeting strategies have utilized simple peptides [106], proteins (including antibodies) or protein fragments [107], carbohydrates, nucleic acids, or vitamins [108, 109, 110, 111, 112].

Both active (ligands-conjugated) and passive (‘non’-conjugated) targeted liposomes are distributed to target cells *via* the same passive distribution mechanism [113]. The field of ligand-targeted liposomes has expanded rapidly despite that several non-targeted liposomes have reached the clinic or in clinical trials [114, 115].

New efforts in targeted drug delivery systems utilize polyunsaturated fatty acids, folic acid, hyaluronic acid, or oligopeptides as tumor recognition moieties. These ligands encounter many discussion fields around their affinity and specificity with no detailed mechanism of tumor-targeting accompanied to limited success for certain small ligands [116], besides the enzymatic degradation in the systemic circulation, making them inappropriate for many *in vivo* studies [117]. Recently, aptamers and aptamer-functionalized nanoparticles high affinity and specificity have great attention in targeted drug delivery systems [118, 119, 120].

Active targeting of the nanocarriers can be achieved through non-covalent or covalent conjugation of targeting ligands to the drug molecule or to the surface of nanocarrier to bind the overexpressed targeting biomarkers selectively on the tumor cells [110, 121]. The direct conjugation of drugs to the targeting ligand can disrupt the receptor/ligand recognition [122] and may alter the drug efficacy [123]. Nano-carrier active targeting enables drugs to be localized within the action site with higher effectiveness [101] to reduce drug dose, minimize the drug side effects and reduced drug variation in blood concentration [124]. Stealth and conventional liposomes usually showed a slow release of the loaded drugs and failed fusion with the endosome after internalization. Consequently, stimuli-responsive liposomes have been introduced to overcome these challenges [125].

#### 2.2.5 Stimuli-responsive liposomes

Stimuli-responsive liposomes are smart liposomal systems that display rapid release of their drug payload upon physicochemical or biochemical stimuli, such as pH, temperature, redox potentials, enzymes concentrations, ultrasound, electric or magnetic fields [126].

Stimuli-responsive liposomes should contain a certain constituent that controls the lipid bilayer's stability and permeability [73]. There are two basic kinds of inductions, remote and local. Remote inductions respond to outside stimuli including, heat, magnetic field, light, electric field, and ultrasounds [73, 125]. Local triggering releases respond to stimuli inside the target tissues, such as pH, redox potential [127, 128], and enzymes [129]. Table 1 represents the most common stimuli-responsive liposomes that respond to specific triggers that lead to a controlled release nanosystem, enhanced intracellular distribution [130].

**Table 1:** The most common stimuli-responsive liposomes.

Stimuli liposomes	Stimuli	Principle	Advantages	Reference
Light-sensitive liposomes	UV, near infrared or visible light irradiation,	Modification of fatty acyl chains of the phospholipids with light-sensitive functional groups and the resulting phospholipids have yielded photoactivable liposomes	Controlling time, exposure, wavelength, and intensity	[131, 132]
Thermosensitive (temperature-sensitive) liposomes	Radiofrequency or microwave ablation	Lipids with a transition temperature of 40–45 °C, such as DPPC, have been employed to make these liposomes	Drug release at high-temperature sites	[133, 134, 135, 136]
Redox-sensitive liposomes	Reactive oxygen species (ROS) peroxides, hydroxyl radicals, singlet oxygen	Depends on the redox potential difference between the intracellular reducing space and oxidizing extracellular space that occur during biological activities.	ROS leads to high concentration levels of glutathione (GSH) in tumor cells cleaving the liposomal formulations	[137, 138]
Enzyme-responsive liposomes	Protease, amidase, and esterase enzymes	Based on amides or esters hydrolysis by protease or esterase enzymes release loaded drugs.	Decreases the adverse side effects of toxic drugs and enable encapsulation of prodrugs	[139, 140, 141]
pH-sensitive liposomes	pH change	Cholesteryl hemisuccinate (CHEMS) and 1,2-dioleoyl-sn-glycero-3-phosphoethanolamine (DOPE), were used to prepare pH-sensitive liposomes	Liposomes with pH-dependent release features	[142, 143, 144]

#### 2.2.6 Bubble liposomes

Bubble liposomes (gas-encapsulated liposomes) are expected to create new applications in the field of gene delivery and drug delivery systems [145]. Recently, liposomes have been used to encapsulate bioactive gases and/or drugs for ultrasound-controlled drug release with enhanced drug delivery [146]. Nitric oxide (NO) bubble liposomes offer a distinguishing NO intravenous therapeutics option overcome common microbubbles, in which liposomes shield NO from hemoglobin rummaging in vitro as usually occurred by free NO. Oxygen bubble liposome (OBL) enables high oxygen fixations with high pO2 conditions of the lungs. This separates OBL from great fluorocarbon and hemoglobin-based oxygen transporters and keeps their utilization as supported oxygen conveyance stages [147].

### 2.3 Methods of preparation

Liposomes can be formulated using different approaches. The process of liposome manufacture and the phospholipids type critically affects the final liposomes characteristics [148]. Liposome's fabrication procedures can be classified into:

#### 2.3.1 Thin film hydration method (Bangham method)

**Figure 8:**
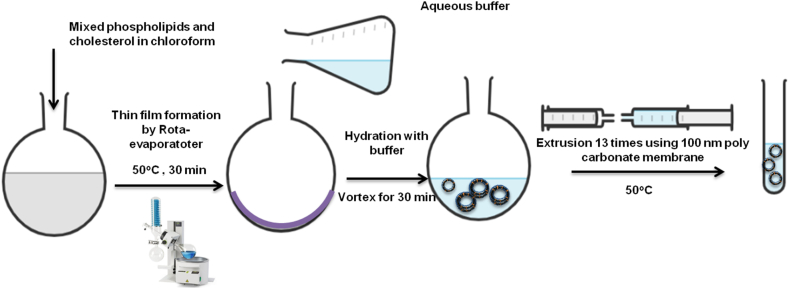
Liposomes preparation *via* thin-film hydration extrusion technique.

In this method, all lipids and the hydrophobic drug are dissolved in suitable organic solvent using a round-bottom flask [50]. The organic solvent then evaporated gently under reduced pressure to create a thin film layer [21]. The obtained thin film is then hydrated, at above the transition temperature (Tm) of the used lipid, with an aqueous buffer solution. The hydration solution may contain a hydrophilic drug/s to be loaded into the liposomes aqueous core. The rate of hydration determines the efficiency of drug encapsulation [148], which the slower the rate of hydration, the higher the encapsulation efficiency [50]. Liposomes resizing, lamellarity types and particles distributions may be controlled by either extrusion through a polycarbonate membranes of specific pore sizes or the use of bath or probe sonicators. Extrusion method ensures stable liposomes with more encapsulation efficiency over sonication. Sonication usually produce SUVs liposomes and may also degrade or hydrolyze encapsulated drugs and/or lipids. Probe sonication may subject liposomes suspensions to potential metal contamination (Figure 8) [21].

#### 2.3.2 Reverse-phase evaporation method

The reverse-phase evaporation method is usually used as an alternative to thin-film hydration by forming a water-in-oil emulsion [148]. First, the lipids are dissolved in an organic solvent which is then directly mixed with an aqueous buffer containing the hydrophilic drug. The organic solvent then evaporated under a reduced pressure rotary evaporator leading to form lipid vesicles dispersed in the aqueous solution. The average size and polydispersity of the preformed vesicles can be reduced by extrusion [149]. This method is suitable for high molecular weight molecules, but therapeutic peptides may be denatured due to organic solvents and to sonication conditions [150].

#### 2.3.3 Solvent injection methods

**Figure 9:**
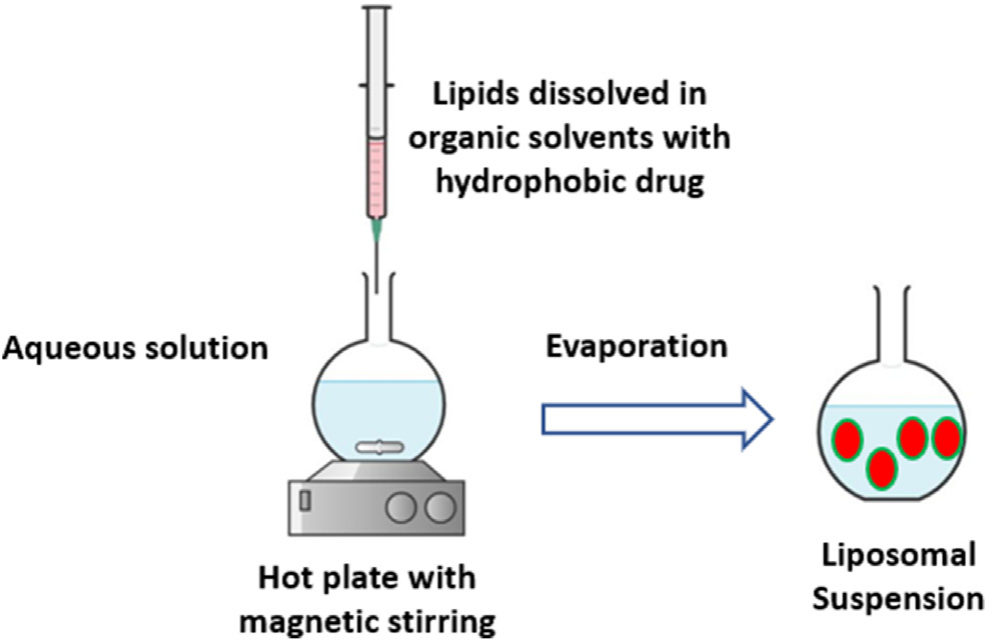
Schematic representation of injection methods method.

The injection methods were classified according to the type of organic solvent used (Figure 9) [151]. An organic solvent dissolving the lipids and the hydrophobic active agents were rapidly injected into an aqueous phase. Diethyl ether enable direct solvent evaporation during mixing process at a temperature above to the boiling point of the used solvent [152]. Utilizing ethanol for injection required a 10-to-20-fold aqueous solution and ethanol can be evaporated under vacuum using a rotary evaporator, dialysis, or filtering. This method mostly prepared liposomal formulations with higher polydispersity indexes (PDI) [153]. In addition, continuous exposure to high temperature and organic solvent might reduce drug and lipids stability [154].

#### 2.3.4 Detergent removal method

In this method, lipids and a high critical micelle concentration (CMC) surfactant were dissolved in a suitable organic solvent using a round bottom flask. A thin film was obtained at the bottom of the flask after solvent gentle evaporation [155]. A mixed micelles solution then obtained by hydrating the lipid film in an aqueous solution containing the drug molecules [156]. The surfactant is then removed by dialysis, size-exclusion chromatography, adsorption onto hydrophobic beads or dilution [157, 158, 159, 160]. Finally, a LUVs liposomes vesicle will be formulated after solution concentration [161]. A main drawback of this method is that most hydrophilic drugs are separated from the liposomes during detergent removal step [162].

#### 2.3.5 Dehydration-rehydration method

It is an organic solvent free method to produce LUVs using sonication. This method based on direct dispersing of the lipids at low concentrations into an aqueous solution containing the drug molecules followed by sonication [163]. First, the dehydration step to evaporate water under nitrogen to create multilayered film entrapping the drug molecules. Then, a hydration step to form large vesicles encapsulating the drug molecules [50, 163]. This method is simple but with high heterogeneity of the liposomes sizes [164].

#### 2.3.6 Heating method

It is also an organic solvent free technique. In this method, lipids are hydrated directly with aqueous solution, and heated for not less than one hour above the Tm of the used phospholipids in the presence of a 3–5 % hydrating agent as glycerin or propylene glycol. The suspension can be heated up to 100 °C when adding cholesterol to the formulation [165]. The hydrating agents act as a stabilizer and isotonizing additives that prevent nanoparticle coagulation and sedimentation. Moreover. The hydration agents provide a cryoprotective effect that makes the heating method an efficient method for the formulation of powder inhalable liposomes [166].

#### 2.3.7 pH jumping method

Another solvent-free method for liposomes preparation is the pH jumping method. In this method, the aqueous solution of phosphatidic acid and phosphatidylcholine are exposed to almost four-fold increase in pH over a short time to break down MLVs into SUVs [167, 168]. The ratio of phosphatidic acid: phosphatidyl choline determine the percentage of SUVs versus LUVs produced [169].

#### 2.3.8 Microfluidic channel method

**Figure 10:**
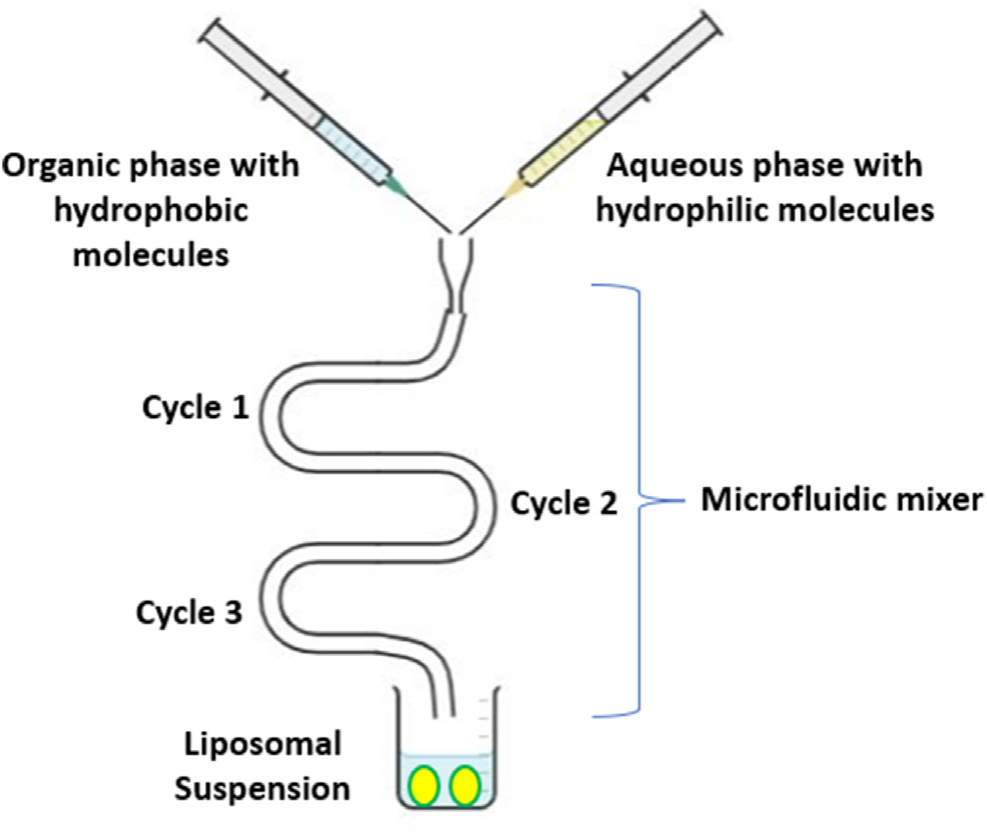
Schematic representation of injection methods method.

The microfluidic channel method (Figure 10) has been recently proposed as a novel method for liposomes preparation. Microfluidics provides a tool to employ liquids within microscopic channels [170]. In this method, lipids are dissolved in ethanol or isopropanol, and the resultant solution is injected upright or in the opposite direction to the aqueous medium within the micro-channels. This method involves continuous axial mixing of the organic and aqueous solutions leads to liposomes formation. Liposomes are stabilized using surfactants to avoid coagulation and separation [171]. Microfluidic channel methods control the mixing process of organic and aqueous phases to achieve reproducible liposomes with proper average size, polydispersity, morphology, and lamellarity [172].

#### 2.3.9 Supercritical fluidic method

This method utilized a supercritical fluid, carbon dioxide (CO_2_), to dissolve lipids instead of using organic solvents. A high-performance liquid pump provides a continuous flow of the aqueous phase into a cell that contains the supercritical lipid solution, allowing phase transition of the dissolved phospholipids [173]. Upon abrupt decrease in pressure, liposomes will formed after completely removing of CO_2_. 5-fold higher encapsulation efficiencies were obtained by this method. This method suffers from high cost, low yield, and special infrastructures even with using the environmentally safe and cheap carbon dioxide [174].

### 2.4 Post preparation handlings

#### 2.4.1 Freeze-thaw cycles

This technique is usually used during liposomes preparations to increase the encapsulation efficiency and to enhance liposome lamellarity. This approach utilized a freeze-thaw cycles between -196 °C in liquid nitrogen and below the transition temperature of the used phospholipids lipids [175, 176].

#### 2.4.2 Freeze-drying (lyophilization)

This treatment is applied to preserve the liposomal products and improve their shelf stability. Freeze-drying involves deep freezing of the liposomes suspension after mixing with a cryoprotective, mainly 5–10 % sucrose or trehalose [177]. Then, a sublimation step at very low temperature and a reduced vacuum was applied to convert the liquid samples to fluffy solid particulates. Lyophilization becomes essential treatment for liposomes encapsulating thermo-sensitive biomolecules [178].

### 2.5 Liposomes characterization

Liposome physiochemical characterization include average size and size distribution (or polydispersity index (PDI)), surface charge (or Zeta potential), shape and morphology, lamellarity, encapsulation efficiency, phase behavior (or polymorphism) and in vitro release profile (Table 2).

**Table 2:** Represent different techniques used for the assessment of liposome parameters.

Liposomes characteristics	Characterization technique	References
Average particle size	Dynamic light scattering (DLS) and microscope technology: Scanning and transmission electron microscopy (SEM/TEM), cryogenic-TEM (Cryo-TEM), and atomic force microscopy (AFM)	[179, 180]
Zeta potential/Surface charge	Electrophoretic mobility, DLS	[181]
Particle shape/morphology	TEM, Cryo-TEM, and AFM	[182]
Lamellarity	Cryo-TEM and ​^31^P-NMR	[182]
Phase behavior	X-ray diffraction (XRD), differential scanning calorimetry (DSC) and thermogravimetric analysis (TGA)	[183, 184]
Encapsulation efficiency/Drug release	Centrifugation, dialysis followed by drug content determination using chromatographic and/or spectrophotometric methods	[185, 186]

### 2.6 Liposomes drug loading

Liposomes drug loading can be attained by passive or active approaches [103]. Passive loading entraps hydrophilic drug in the liposomes aqueous core during lipid bilayer formation, while hydrophobic drugs accumulate in the small-sized hydrophobic lipid bilayer [103, 187, 188, 189]. Passive loading suffers from bilayer destabilization, high drug/lipid ratio, and rapid drug release [101]. Therefore, improving the aqueous solubility of these hydrophobic drugs by cyclodextrin host-guest complexation were successfully applied and permitliposomes aqueous core loading by forming drug-in-cyclodextrins-in-liposomes delivery system [190].

Active or remote loading has been developed to ensure high encapsulation efficiency of precious chemotherapeutic agents [191]. Remote loading can be achieved into preformed liposomes by pH gradient and/or potential ionic differences across liposomal bilayer membranes [101,187, 192]. The success of intraliposomal remote loading are govern by to main parameters, (i) drug aqueous solubility (ii) presence of an ionizable functional group in drug chemical [192, 193, 194].

Intraliposomal active loading of hydrophobic drugs in response to ionic and/or pH gradients across the liposomes bilayer was developed [194, 195]. This procedure enables hydrophobic drugs to accumulate inside the liposomes core after the vesicles are created. The advantage of this method is that the loading of the drug can be performed independently of liposomes preparation conditions [101]. Most potentially active drugs are weak bases possessing primary, secondary, or tertiary amine functional groups that can be loaded in response to pH gradients [196]. Drugs that are not weak bases, or do not have an ionizable functional group, can be converted to weak base prodrugs or encapsulated with amino-modified carriers as cyclodextrins, therefore allowing encapsulation and intraliposomal retention [193, 197, 198].

## 3 Protein corona fingerprints of liposomes

Liposomes have been used to overcome many problems associated with low efficiency of anticancer drugs [199]. Recently, a concept is emerging that the limited success of liposomal drugs in clinical practice due to poor knowledge of liposomes behavior in vivo. Lipid vesicles are usually covered by plasma proteins in vivo forming a biomolecular coating, referred to as the protein corona (PrC) [200]. Recent studies verified that PrC fingerprints (PrCFs) enhanced liposome attachment with cancer cells, triggering efficient particle localization and internalization [201].

Accordingly, enrichment in PrCFs was utilized to predict the targeting ability of synthesized liposomal formulations. Palchetti et al reported that the targeting capability of liposome–protein complexes clearly relate with cellular uptake in pancreatic adenocarcinoma (PANC-1) and insulinoma (INS-1) cells as quantified by flow-assisted cell sorting (FACS). The results showed that cellular uptake of the liposomal formulation with the highest abundance of PrCFs was much larger than that of Onivyde®, an Irinotecan liposomal drug approved by the Food and Drug Administration in 2015 for the treatment of metastatic Pancreatic ductal adenocarcinoma (PDAC) [201]. Furthermore, Digiacomo et al identified a potential protein biomarker for pancreatic ductal adenocarcinoma (PDAC) by utilizing liposomes to accumulate PrC coating layer from human plasma proteins. These targeting liposomes may be used for the early diagnosis of PDAC [202]. This approach could open the interesting possibility to identify novel biomarkers for liposomes formulations in the context of personalized medicine.

## 4 Liposomes in clinical applications

Various liposomal-based formulations were successfully implemented in clinical fields as antitumor, anti-fungal therapies, analgesics [203]. Doxil® was the first approved clinical anticancer liposome drug in the USA (1995). It opened the way to several other liposomal formulations to get to the clinical application fields by innovating the pH gradient active loading and usage of PEGylation for stealth liposomes [203, 204]. Conventional liposome without PEGylation, can be attractive when circulation half-life is not the goal [205]. DepoFoam™ is mostly used for gradual drug release, thus maintaining a continuous drug supply for long-lasting effect [206].

### 4.1 Marketed clinical liposomes

#### 4.1.1 Cancer treatment

Doxil® or Caelyx® was presented in 1995 by Sequus Pharmaceuticals. Doxil was designed as a polyethylene glycol coated doxorubicin (DOX) liposome intended for the treatment of Kaposi's sarcoma [204]. LipoDox® is another FDA approved PEGylated liposomal formulation encapsulating DOX manufactured by Sun Pharma in 2012 [207]. Daunorubicin was the second anthracycline antineoplastic drug loaded in liposomes to treat acute myeloid leukemia (AML) under the generic name DaunoXome® [208]. Myocet® is a non-PEGylated liposomes encapsulating DOX that showed a shorter circulation half-life with less cardiac side effects [205,209].

Depocyt® consists of Citarabine, a cell-cycle cytotoxic drug, enclosed in the DepoFoam™ multivesicular enclosure, which allows a sustained two-week release [210]. A new liposomes formulation called Mepact® was globally approved for the treatment of osteosarcoma [211]. Vincristine also incorporated into sphingomyelin/cholesterol-based liposome under the name of Marqibo®. This approved formula offered longer circulation time without surface-modified, resulting in a higher accumulation in target tissues in which vincristine is gradually released [212]. Onivyde® is another PEGylated liposome carrying irinotecan and exhibits a long-acting, antitumor effect [213]. In addition, Vyxeos® also known as CPX-351, is composed of a combination of Cytarabine and daunorubicin, encapsulated in a liposome in a ratio of 5:1. This formulation reduced adverse effects with enhanced effectiveness [213, 214]. Finally, Paclitaxel, an anticancer drug, was also incorporated into Lipusu® liposomes to treat gastric carcinoma efficiently with much less adverse effects [215].

#### 4.1.2 Fungal treatment

A two major approved anti-fungal liposomes formulation were Ambisome® and Fungisome®. They encapsulate Amphotericin B anti-fungal drug with many advantages compared free drug [216, 217]. These Amphotericin B liposomes were stabilized in saline and have longer bioavailability and less toxicity and side effects [218, 219].

#### 4.1.3 Photodynamic therapy

Visudyne®: is the only liposomal drug delivery agent approved for age-related macular degeneration therapy by inhibiting the generation of blood vessels in the eye [220].

#### 4.1.4 Pain management

DepoDur™ is a morphine formulation using DepoFoam™ Technology that resulted in a sustained release formula with prolonging the clinical effect time [206]. Exparel® also uses the DepoFoam™ technology to release Bupivacaine for sustained pain relief [197] gradually.

Table 3 summarizes the different clinically approved liposomes formulations in terms of their purpose, lipid constituents, active ingredients, and administration route.

**Table 3:** Clinically used liposomes grouped by therapeutic usage.

Usage	Trade name	Active ingredient(s)	Liposome platform (Molar Ratio)	Manufacturer	Year Approved	Administration Route	References
Anti-Cancer	Doxil®	Doxorubicin	HSPC:Cholesterol:PEG 2000-DSPE (56:38:5)	Sequus Pharmaceuticals	1995	I.V	[204]
	DaunoXome®	Daunorubicin	DSPC:Cholesterol (2:1)	NeXstar Pharmaceuticals	1996	I.V	[208]
	Depocyt®	Cytarabine	DepoFoam™	SkyPharma Inc.	1999	Spinal	[210]
	Myocet®	Doxorubicin	Cholesterol:EPC (45:55)	Elan Pharmaceuticals	2000	I.V	[209]
	Mepact®	Mefamurtide	DOPS:POPC (3:7) Multilamellar liposome	Takeda Pharmaceutical Limited	2004	I.V	[211]
	Lipodox®	Doxorubicin	DSPC:Cholesterol:PEG 2000-DSPE (56:39:5)	Sun Pharma	2012	I.V	[205, 207]
	Marqibo®	Vincristine	SM:Cholesterol (60:40)	Talon Therapeutics	2012	I.V	[212]
	Onivyde™	Irinotecan	DSPC:Cholesterol:MPEG-2000-DSPE (3:2:0.015)	Merrimack Pharmaceuticals	2015	I.V	[213]
	Lipusu®	Paclitaxel	NA	Luye Pharma Group	2006	I.V	[215]
	Vyxeos®	Cytarabine:Daunorubicin 5:1	DSPC:DSPG:Cholesterol (7:2:1)	Jazz Pharmaceuticals	2017	I.V	[214, 221]
Anti-Fungal	Ambisome®	Amphotericin B	HSPC:Cholesterol:DSPG (2:1:0.8)	Astellas Pharma	1997	I.V	[216]
	Fungisome®	Amphotericin B	PC:Cholesterol (7:3)	Lifecare Innovations	2003	I.V	[219]
Photodynamic therapy	Visudyne®	Verteporphin	Verteporphin:DMPC&EPG (1:8)	Novartis AG	2000	I.V	[220]
Analgesic	DepoDur™	Morphine sulfate	DepoFoam™	SkyPharma	2004	Epidural	[206]
	Exparel®	Bupivacaine	DepoFoam™	Pacira pharmaceuticals	2011	I.V	[222]

### 4.2 Liposomes in clinical trials

**Figure 11:**
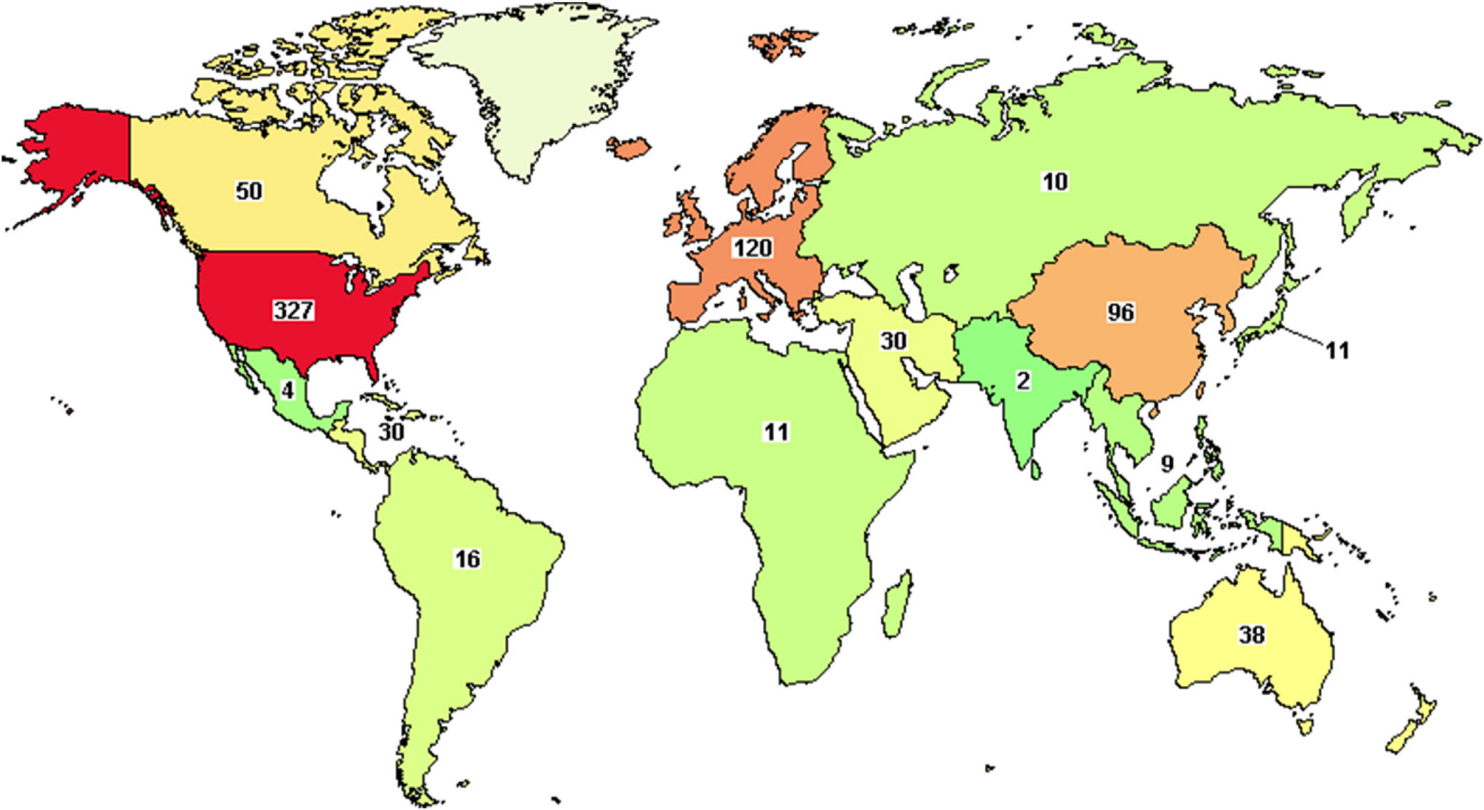
Active clinical trials as per 28/dec/2021, source: https://ClinicalTrials.gov.

From the 83316 active clinical trials registered, 511 liposomal clinical trials investigating liposomal products which are distributed worldwide as shown in Figure 11. The drugs being examined belong to anticancer drugs, analgesics, immune-modulators, anti-fungal, etc. Among these drugs, 121 of the 511 are in phase III testing, 236 are in phase II, 120 are in phase I, and 6 in early phase I [20].

### 4.3 Liposomes in vaccinations

Liposome formulations could protect DNA/RNA and proteins payload from biodegradation. Furthermore, their transfection efficiency could be enhanced by modifying surface charge, size, and lipid structure. Two commercial vaccines based on virosome technology are currently on the market, Epaxal® and Inflexal® V (Berna Biotech Ltd, Bern, Switzerland), a hepatitis A vaccine. Virosomes are liposomal formulations that have viral envelope proteins anchored to their lipid membrane [223].

Recently, COVID-19 mRNA based-vaccines utilized liposomes protection to increase their in vitro and in vivo stability [224]. Liposome-based mRNA anti-COVID-19 vaccine has been designed by Pfizer/BioNTech and Moderna, and already administered worldwide. These vaccines were made to maintain the stability of liposomes in blood and to promote immune responses. Their components include distearoyl phosphatidyl choline and cholesterol that considered the main constituents of conventional liposomes [225]. Four major ingredients were used in COVID-19 vaccines: Cationic lipids, for instance 1,2-dioleoyl-3 (trimethylammonium) propane (DOTAP), to binds to the negatively charged mRNA, pegylated lipids stabilize the particle, phospholipids and cholesterol molecules that form the required structure [226]. These formulas encapsulate mRNA, protect it from nucleases, and deliver it into cells, where the mRNA is released and used to generate proteins. During COVID-19 pandemic, many liposome-based vaccines have been developed with great success. Accordingly, mRNA coding for the protein spike of the Coronavirus, would be encapsulated into liposomes that are designed to be stable in the circulating blood until they are taken up by phagocytic cells in the body by endocytosis. The mRNA will then be expressed as the spike protein in turn promoting an immune response to it that will kill or inactivate the invading virus [224].

## 5 Conclusion

Liposomes were successfully utilized as an efficient drug delivery system for various diseases ranging from cancer treatment to pain managing. The biocompatible, biodegradable, and low immunogenicity liposomes formulation enhanced the pharmacokinetics and pharmacodynamics properties of water insoluble, poor bioavailable and highly toxic drug. Liposomes undergone numerous evolutions in terms of their constituents and manufacturing process to overcome their early limitations. Several liposomes formulation is currently approved in the market to treat various diseases and more than five hundred liposomal formulations are now in different phases of clinical investigation. Nevertheless, liposomes critical challenges are their physical and chemical stability. As a result, there are a n essential need to develop liposomes with high stability significantly impacts their clinical application. Thus, in silico simulation and computational investigations may enable approximate estimation for the best liposomal formulation in their constituents and 3-D structure morphology.

## Declarations

### Author contribution statement

All authors listed have significantly contributed to the development and the writing of this article.

### Funding statement

This research did not receive any specific grant from funding agencies in the public, commercial, or not-for-profit sectors.

### Data availability statement

No data was used for the research described in the article.

### Declaration of interests statement

The authors declare no conflict of interest.

### Additional information

No additional information is available for this paper.
